# Rapid targeted gene disruption in *Bacillus anthracis*

**DOI:** 10.1186/1472-6750-13-72

**Published:** 2013-09-18

**Authors:** Roland J Saldanha, Adin Pemberton, Patrick Shiflett, Jiri Perutka, Jacob T Whitt, Andrew Ellington, Alan M Lambowitz, Ryan Kramer, Deborah Taylor, Thomas J Lamkin

**Affiliations:** 1 UES Inc, 4401 Dayton-Xenia Road, Dayton, OH 45432, USA; 2 Henry Jackson Foundation, 6720-A Rockledge Drive, Suite 100, Bethesda, MD 20817, USA; 3 Institute for Cellular and Molecular Biology, The University of Texas at Austin, Austin, TX 78712-0159, USA; 4 Air Force Research Laboratory, Air Force Research Laboratory, 711th HPW/RHXBC, Molecular Signatures Section, 2510 Fifth Street, Area B, Bldg 840, Room W220, Wright-Patterson AFB, OH 45433-7913, USA; 5 Department of Molecular Genetics, Biochemistry and Microbiology, University of Cincinnati College of Medicine, Cincinnati, OH 45267-0524, USA

## Abstract

**Background:**

Anthrax is a zoonotic disease recognized to affect herbivores since Biblical times and has the widest range of susceptible host species of any known pathogen. The ease with which the bacterium can be weaponized and its recent deliberate use as an agent of terror, have highlighted the importance of gaining a deeper understanding and effective countermeasures for this important pathogen. High quality sequence data has opened the possibility of systematic dissection of how genes distributed on both the bacterial chromosome and associated plasmids have made it such a successful pathogen. However, low transformation efficiency and relatively few genetic tools for chromosomal manipulation have hampered full interrogation of its genome.

**Results:**

Group II introns have been developed into an efficient tool for site-specific gene inactivation in several organisms. We have adapted group II intron targeting technology for application in *Bacillus anthracis* and generated vectors that permit gene inactivation through group II intron insertion. The vectors developed permit screening for the desired insertion through PCR or direct selection of intron insertions using a selection scheme that activates a kanamycin resistance marker upon successful intron insertion.

**Conclusions:**

The design and vector construction described here provides a useful tool for high throughput experimental interrogation of the *Bacillus anthracis* genome and will benefit efforts to develop improved vaccines and therapeutics.

## Background

In 1876 Robert Koch ushered in microbial pathology by establishing a causal relationship between *Bacillus anthracis* microbes and the disease anthrax [1]. Distributed worldwide, *Bacillus anthracis* is a gram positive bacterium capable of infecting the widest range of animals of any known pathogen [2]. The bacterium is normally found as an endospore in soil where a chance encounter with an animal can permit entry through a wound, food or inhalation leading to germination of the spore into a vegetative bacterium. The vegetative phase is relatively short consisting of only 20–40 generations. The bacterium is believed to spend large periods of time in spore form thus slowing evolutionary changes and explaining the monomorphic nature of the bacterium [3]. Human infection occurs most commonly through accidental exposure to infected animals or animal products, such as hides or carcasses. 95% of infections occur via the cutaneous route and more rarely through gastrointestinal and pulmonary routes [4,5]. The disease is difficult to diagnose because early symptoms are relatively non-specific and, when left untreated without prompt antibiotic intervention, can lead to fatality with rates ranging from 20% for cutaneous exposure to as high as 100% for pulmonary anthrax. The high morbidity is due to a rapid overwhelming of host defenses, fulminant septicemia of vegetative cells and the action of bacterial toxins expressed from vegetative cells. Once inside the host, the spores are engulfed by macrophages where they are transported to lymph nodes and germinate to become vegetative cells that eventually disseminate through blood and lymph causing septicemia and toxemia. The vegetative cells express three monomeric proteins: protective antigen (PA), lethal factor (LF) and edema factor (EF) from genes encoded on the pXO1 virulence plasmid [6]. PA facilitates toxin entry by binding to receptors TEM8 and CMG2 on the surface of human and animal cells. Binding triggers PA cleavage leading to formation of a heptameric or octameric pore that binds and translocates EF and LF into the cytosol. In binary complexes with PA, EF and LT are referred to as edema toxin (ET) and lethal toxin (LT) respectively. ET is an adenyl-cyclase that enhances cAMP production and disrupts water homeostasis leading to kidney failure and death. LT is a zinc protease that cleaves mitogen-activated protein kinase kinases (MAPKK) and induces pro-inflammatory cytokines and cellular apoptosis [4]. Antibodies predominantly directed against PA are the basis of current vaccines that are used prophylactically [7].

The dramatic lethal consequences of an accidental spore release at Sverdlovsk in the former Soviet Union and the deliberate release of anthrax through letters in the US in 2001 have renewed interest in neutralizing this pathogen [8,9]. The complete sequence of *Bacillus anthracis* and several of its close relatives is available [10,11]. This potentially allows for systematic study of all the genes contributing to the success of this important pathogen. However, a complete dissection of *Bacillus anthracis* is hampered by the difficulty of transforming the bacteria (approximately 10^2^-10^5^ transformants per μg DNA) and by relatively few simple ways to manipulate the genome genetically [12-15]. Shatalin and Neyfakh used a temperature sensitive (*ts*) plasmid to deliver a selectable marker flanked by upstream and downstream regions homologous to the gene to be disrupted [14]. Through the manipulation of temperatures and screening of antibiotic markers, a chromosomal integrant could be obtained. Janes and Stibitz have created an elegant set of plasmids to make markerless gene disruptions in two distinct steps [12]. In the first step, a plasmid is constructed with an I-SceI site flanking a genetic marker to be replaced and delivered on a *ts* replicon using an erythromycin selection marker. On introduction of this plasmid in *Bacillus anthracis*, the plasmid can integrate into the chromosome at the low frequency of homologous recombination using the homology of genomic sequences cloned into the plasmid. On shifting to a restrictive temperature the plasmid is cured and it is possible to select for those rare chromosomal integration events. After appropriate steps to verify the success of the first step, a second plasmid expressing I-SceI is introduced. This leads to cleavage at the I-SceI site and stimulating recombination via double strand break repair which either leads to the desired recombination product or restoration to the parental chromosomal configuration. The desired product must be obtained through physical screening of individual colonies. Finally, Leppla and colleagues have exploited a selectable marker flanked by the *loxP* sites of the site-specific recombinase Cre to leave a single *loxP* site at the site of marker integration which could then direct larger genome scale deletions through multiple rounds of *loxP* marker insertion at distant sites and site-specific recombination between them [13].

Many group II introns exhibit the ability to insert into DNA through a now well understood series of biochemical reactions [16]. The natural mobility of the Ll.LtrB intron has been harnessed into a highly efficient gene targeting technology to achieve gene disruption through intron insertion in a number of gram positive and gram negative bacterial hosts [17,18]. The ability to engineer a group II intron to insert at desired genomic locations, distinguishes group II introns from other insertional elements like transposons that either integrate randomly at a low frequency or insert at a high frequency but only at a specific genomic locus like the *att*Tn7 site. Frequently, the engineered group II introns insert at their newly programed insertion sites in the genome at frequencies high enough to be detectable by simple colony screening. The high efficiency of insertion allows gene disruption without the introduction of an antibiotic marker into the chromosome and the efficient sequential creation of multiple knockouts at disparate chromosomal sites. We have adapted the Ll.LtrB intron insertion technology for application to *Bacillus anthracis* and demonstrate the remarkable simplicity with which gene inactivation through intron insertion can be rapidly achieved.

## Results and discussion

### Group II intron based gene inactivation

Group II introns in general, and the Ll.LtrB group II intron in particular, have been engineered into an efficient gene disruption system [17,19,20]. We have adapted this system for rapid gene inactivation in *Bacillus anthracis*. Group II introns are autocatalytic RNA molecules [21]. Some group II introns exhibit a property of “homing” where the intron recognizes the DNA equivalent of its cognate exon junctions and inserts itself via retrotransposition [16]. The biochemical basis of this intron insertion has been deduced [22-24]. For example, the Ll.LtrB intron interrupting a relaxase in *Lactococcus lactis* is excised from its flanking exons through RNA catalysis assisted by an intron encoded protein LtrA [25]. The LtrA protein acts as a scaffold for efficient RNA directed catalysis but also encodes a reverse transcriptase and DNA endonuclease that are used for intron integration into DNA targets [26]. The spliced Ll.LtrB intron remains associated with LtrA to form a RNP particle. The intron component of the RNP can recognize DNA exon sequences resembling exon junctions through a combination of exon recognition elements (EBS1/δ and EBS2) within the intron and DNA recognition preferences of the LtrA protein [27]. On encountering a suitable target, the RNA component of the RNP can integrate through reverse splicing of the intron RNA into the DNA target, followed by an LtrA directed cleavage of the bottom DNA strand which generates a primer that allows copying of the intron into DNA via the reverse transcriptase activity of LtrA.

### A vector permitting group II intron gene inactivation using screening

**Figure 1:**
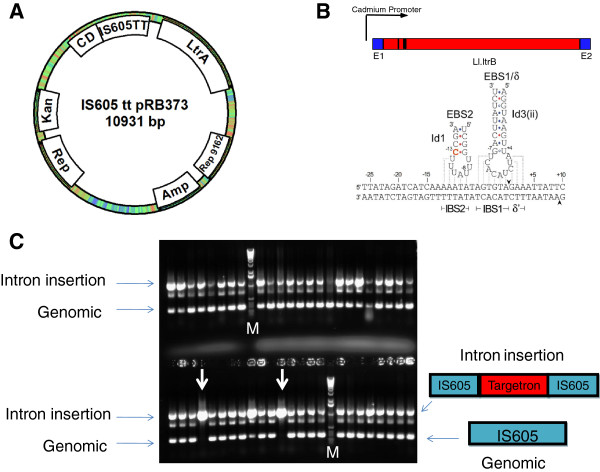
**Bacillus anthracis Targetron plasmid. (A)** A variant of the Ll.LtrB group II intron designed to insert into orfB of IS605 elements of *Bacillus anthracis* was cloned into a *Bacillus anthracis/E. coli.* shuttle vector with a kanamycin resistance marker (bacillus) and ampicillin marker for *E. coli*. The intron and intron encoded LtrA protein are driven from a Cadmium inducible promoter (Cd). **(B)** EBS1/δ and EBS2 (shown schematically as black bars in the intron) are sequences within the intron controlling exon recognition via base pairing interactions. Reprogrammed sequences in EBS1/δ and EBS2 for efficient integration into IS605 are shown. **(C)** Plasmid IS605tt pRB373 was introduced into *Bacillus anthracis* Sterne via electroporation and selection for kanamycin resistant colonies. PCR screening of individual colonies using primers flanking the potential site of intron insertion show virtually all colonies had an intron insertion. There are 3 genomic copies of IS605 and in the two lanes highlighted by a white arrow all three copies have an intron insertion. Lanes marked M have 1 kb molecular weight markers.

To adapt group II introns for targeted gene disruption in *Bacillus anthracis* we cloned the Ll.LtrB intron and LtrA protein under the control of a cadmium inducible promoter [28] using a gram positive/gram negative shuttle vector pRB373 as a backbone [29]. This vector offers a pUB110 origin of replication for gram positives and a ColE1 origin for gram negatives. The selection markers are kanamycin/neomycin in gram positives and ampicillin in gram negatives. For proof of principle, we changed the sequences in EBS1/δ and EBS2 within the intron to direct the intron to orf B of the IS605 insertion sequences which has 3 copies in the *Bacillus anthracis* genome (located in ORF 1652, ORF 2876 and ORF 5071). Figure 1A shows general features of the vector and the modified base pairing interactions within EBS1/δ and EBS2 that allow the intron to recognize and insert itself into IS605 orf B which are shown in Figure 1B. The plasmid shown in Figure 1A was introduced into *Bacillus anthracis* Sterne by electroporation and kanamycin resistant colonies were obtained. A single colony was grown overnight and induced with 10 μM cadmium for 90 minutes. Dilutions were plated on LB without antibiotic selection and individual colonies were screened for presence of the intron insertion using PCR primers flanking the insertion site. Figure 1C shows that this intron is remarkably efficient, and that every colony screened yielded a PCR product consistent with intron insertion into orf B of IS605. Direct sequencing of the PCR product confirmed that the intron did successfully integrate at the desired locus. Figure 1C also shows that in most cases a PCR product that is consistent with an intron free genomic product is also detectable, which suggests that the intron has not integrated at all three possible insertion sites. In two cases (highlighted by a white arrow) a single PCR product is seen which is consistent with intron insertion in all three potential insertion sites. The remarkable efficiency of this intron represents the upper end of intron targeting efficiency [19]; i.e. 100% insertion efficiency for all colonies screened with 1 in 24 colonies showing an intron insertion into three independent chromosomal insertion targets. For other targets we have examined, the intron efficiency is considerably lower requiring extensive arduous PCR screening to obtain a single insertion.

### A vector using genetic selection for detection of group II intron gene insertion

**Figure 2:**
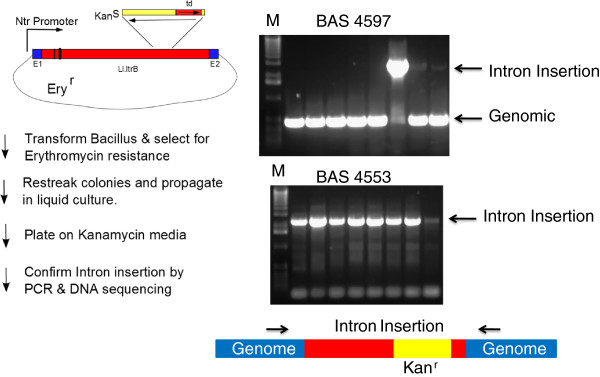
**Bacillus vector for selection of group II intron insertions.** A schematic of the important vector features and selection scheme for group II intron based gene disruption. The plasmid is erythromycin resistant but kanamycin sensitve because a *td* group I intron interrupts its coding sequence in an antisense orientation. Transcription of the group II intron from the *Ntr* promoter allows self- splicing of the *td* intron and generates a substrate that is capable of genomic integration at pre-determined loci due to changes in sequences shown as black bars within the intron. Genomic integrants no longer have the *td* group I intron interrupting the kanamycin resistance gene and thus successful integrants are kanamycin resistant. For PCR verification of intron insertion, primers (shown as arrows above the genomic DNA schematic) were designed to flank the site of intron insertion in BAS4597 and BAS4553. kanamycin resistant colonies arising in *Bacillus anthracis* Sterne after transformation of plasmids reengineered to integrate the group II intron at BAS4597 and BAS4553 were screened by colony PCR and analyzed after electrophoresis on a 1% agarose gel in TBE buffer and visualized by staining with ethidium bromide. Lanes marked M have 1 Kb molecular weight markers.

To address the problem of finding intron insertions for less efficient introns, we have engineered a vector with a retrotransposition activated kanamycin resistance marker that allows selection of intron insertion events [30]. Figure 2 illustrates the main features in this vector. An erythromycin resistance marker was introduced into a pRB373 shuttle vector. Group II intron expression was driven using an Ntr promoter previously identified by using a promoter trap strategy [31]. The kanamycin resistance marker that is conditionally expressed only after intron insertion was created by total DNA synthesis and used codons optimized for low GC organisms. This marker is expressed under its own promoter and is cloned at a known neutral site in domain IV of the intron in an antisense direction relative to the group II intron expression. A *td* self-splicing group I intron interrupts the kanamycin resistance coding sequences in the same orientation as the Ll.LtrB group II intron. As a result of this configuration, the kanamycin resistance marker in the donor plasmid is silent. Any leak-through expression from its own promoter leads to a non-functional protein since the group I intron interrupting it cannot be spliced out due to its antisense orientation relative to kanamycin expression. Group II intron RNA transcripts will, however, also have the *td* intron autocatalytically removed restoring the continuity of the kanamycin coding sequence. If these transcripts are used as substrates for site-specific intron integration through reverse splicing into DNA targets and reverse transcription, the kanamycin marker can then be expressed. Thus successful retrotransposition events are marked by a gain of kanamycin resistance which allows for selection of rare intron integration events. As a proof of principle, we have designed introns to insert at BAS4553 (a methionine gamma-lyase) and BAS4597 (a pullulanase). The regions within the intron that interact with DNA insertion sites were changed in order to direct the intron to insert between nucleotides 343:344 of the BAS4553 gene as well as between nucleotides 1794:1795 of the BAS4597 gene. The reengineered plasmid vectors were introduced into *Bacillus anthracis* Sterne by electroporation and selection on erythromycin plates. Transformants were regrown in liquid media containing erythromycin and plated on LB agar with kanamycin to select for putative intron integration events. Kanamycin resistant colonies were screened for successful intron insertion by using PCR primers that flanked the site of intron insertion. As can be seen in Figure 2, all kanamycin resistant colonies for BAS4533 show a PCR product consistent with group II intron insertion. For BAS4597, only one of the 8 colonies is consistent with intron integration at the desired site. The PCR products were sequenced and indicate precise intron integration at the desired locus.

## Conclusions

The vectors described here introduce a new tool for genetic manipulation of *Bacillus anthracis*. The ability to directly select for an insertional inactivation of a gene using very few manipulations will accelerate our ability to probe the role of the numerous uncharacterized genes in the genome of this important pathogen and uncover new targets for vaccination strategies or useful targets for therapeutic intervention. While the vectors were developed and tested in *Bacillus anthracis*, the plasmid backbone used to build the vector is derived from pUB110 a *Staphylococcus aureus* plasmid that has been extensively used in *Bacillus subtilis*[29]. These vectors are therefore likely to be able to be used across the *Bacillus* genus including other members of the *Bacillus cereus* group such as *Bacillus cereus* and *Bacillus thuringiensis*.

## Methods

### Bacterial strains and culture

*E. coli* strains: DH5α™ with genotype F– Φ80lacZΔM15 Δ(lacZYA-argF) U169 recA1 endA1 hsdR17 (rK–, mK+) phoA supE44 λ– thi-1 gyrA96 relA1 and *dam*^*-*^*dcm*^*-*^ (Life Technologies) was used for routine cloning. Since transformation efficiency of *Bacillus anthracis* is sensitive to dam methylation [32], plasmid DNA for transformation into *Bacillus anthracis* was prepared from a dam methylase deficient host *E. coli* (New England Biolabs C2925H) with genotype: ara-14 leuB6 fhuA31 lacY1 tsx78 glnV44 galK2 galT22 mcrA dcm-6 hisG4 rfbD1 R(zgb210::Tn10) Tet^S^ endA1 rspL136 (Str^R^) dam13::Tn9 (Cam^R^) xylA-5 mtl-1 thi-1 mcrB1 hsdR2.

*Bacillus anthracis* (Sterne 34 F2) was obtained from Colorado Serum Co. (Denver, CO).

Bacteria were grown in Luria-Bertaini (LB) broth or Brain Heart Infusion (BHI). Antibiotics were added for plasmid maintenance or selection at the following concentrations: ampicillin at 100 μg/mL; erythromycin at 400 μg/mL for *E. coli* and 4 μg/mL for *Bacillus anthracis* and kanamycin was used at 50 μg/mL for *E. coli* and 20 μg/mL for *Bacillus anthracis.*

### Plasmid construction

The pRB373 plasmid was purchased from ATCC (cat. no. 77373). The sequence of pRB373 is unpublished but based on restriction enzyme digestion it contains a BsrG1 site. Because of this, a construct designed to retarget the Ll.LtrB intron to insert within IS605 needed to be created in three steps. In the first, a PCR fragment containing the changes necessary to retarget the intron to IS605 was digested with BsrGI and HindIII and cloned into the intron donor plasmid pNL9161 of *Staphylococcus aureus* digested with the same enzymes [33]. To optimize pairing at the +1 site of the target, this intermediate plasmid was modified by mutating C + 1 to G + 1. The resulting plasmid was then digested with SphI and SfoI, and the fragment containing the Cd-inducible promoter, LtrB intron, and LtrA protein was gel purified and cloned between the SphI and SmaI sites of pRB373 to yield plasmid IS605-tt.

Preparation of plasmid DNA from *E. coli*, transformation of *E. coli*, and recombinant DNA techniques were carried out by standard procedures [34]. The plasmid pRBEryNtr was constructed in several steps. In the first step an erythromycin resistance marker was PCR amplified using pNL9162 as the template and oligonucleotide primers SphI Ery 5’ GAATGAGGCATGCTACACCTCCGGATA3’ and AgeI Ery 5’ GAT C ACCGGTCACACGAA AAACAAGTTAAGGGATGCAG3’ using NEB PCR mastermix. This yielded a 1.2 Kb fragment which was then purified and digested with SphI and AgeI. It was then cloned into pRB373 (which had been digested with the same enzymes) to yield pRB373Ery. The potent Bacillus *Ntr* promoter identified by Gat et al., [31] was amplified from *Bacillus anthracis* Sterne genomic DNA using PCR primers 5’ SphI Nitro 5’ GatC GCATGC CTGAGTTGGATCATCATTATATGAAAGGC3’ and 3’ Nitro HindIII XhoI EcoRI 5’Gatc gaattc ctcgag gag AAGCTTTTTTTCA TATGTATAC ATC ATA TTC TGC C3’. The PCR product was digested with SphI and EcoR1 then cloned into pRB373Ery digested with the same enzymes to yield pRB373 EryNtr. The coding sequence of a select agent compliant kanamycin allele was optimized for expression in low GC organisms and synthesized (Genscript, Piscataway, NJ). The synthetic fragment also included a self-splicing *td* intron in the antisense orientation inserted between codons 15 and 16 of the kanamycin gene and cloned into pRB373. A HindIII-XhoI fragment with the group II intron Targetron was cloned into pRB373 digested with the same enzymes. Finally, PCR mutagenesis was used to generate the mutations necessary to retarget the intron to BAS4597 & BAS4553 and introduced as HindIII-BsrG1 fragments into the vector as described above.

### Intron design & generation of PCR products for intron retargeting

The sequence of *Bacillus anthracis* genomic DNA to be targeted for disruption was scanned for potential intron insertion sites using a computer algorithm derived from a learning set of successful intron integrations [19]. The resulting insertion sites are shown in Table 1 and the mutations necessary to retarget the intron to a desired site were introduced through PCR mutagenesis using two pairs of partially overlapping primers (Table 2) to assemble a short DNA fragment flanked by HindIII and BsrG1 sites and incorporating the changes within the intron required to redirect the intron to a new locus as described previously [17]. Briefly, for each mutant intron to be created 10 cycles of PCR was performed using 2 μL of the mutant specific IBS and EBS universal primer at a concentration of 100 μM and 10 μM respectively in a 20 uL reaction using Phusion^®^ (NEB) PCR mastermix and 0.2 ng of wild-type intron template. In a separate tube a similar reaction was performed using mutant specific primers EBS1delta (100 μM) and EBS2 (10 μM) primers. At the end of the first round of asymmetric PCR, the two reactions were combined and allowed to proceed through 20 more PCR amplification cycles. The resulting PCR products incorporating all necessary mutations were gel purified, digested with HindIII and BsrG1 and cloned into the intron donor plasmids described above cut with the same enzymes.

**Table 1:** Targetron insertion sites and scores

**Gene insertion site**	**-30 to -1 NT upstream**	**LtrB intron**	**+1 to +15 NT downstream**	**Score**
IS605 435|436 s	CCGATTATAGATCATCAAAAATATAGTGTA	gtgc…tcac	GAAATTATTCGCAAA	9.3
BAS4553 343|344 s	GTTCAAATGGATTATACGGATGCACGTACG	gtgc…tcac	GCTTTTTGGAAGTGT	8.4
BAS4597 1794|1795 s	AATCAATTAGATTGGGATCGAAAAGAGAAA	gtgc…tcac	GAAATAGAGACCGTT	7.3

The gene chosen for intron insertion is shown in the first column with the nucleotide positions at which the intron is designed to insert at within the gene shown beside the gene name. All intron insertions are in the sense orientation relative to gene transcription and designated here with the suffix “s”. The sequence of 30 nucleotides upstream and 15 nucleotides downstream of the intron insertion site are shown together with a numeric score reflecting expected efficiency based on a learning set of Ll.Ltrb intron insertions.

**Table 2:** Primers used for retargeting

**Primer name**	**Primer sequence**	**Pairings changed**
IS605-435|436 s	AAAAAAGCTTATAATTATCCTTAAAATACAGTGTAGTGCGCCCAGATAGGGTG	IBS1/2
IS605-435|436 s	CAGATTGTACAAATGTGGTGATAACAGATAAGTCAGTGTAGATAACTTACCTTTCTTTGT	EBS1/δ
IS605-435|436 s	TGAACGCAAGTTTCTAATTTCGGTTTATTTCCGATAGAGGAAAGTGTCT	EBS2
BAS4553-343|344 s	AAAAAAGCTTATAATTATCCTTAGATGCCCGTACGGTGCGCCCAGATAGGGTG	IBS1/2
BAS4553-343|344 s	CAGATTGTACAAATGTGGTGATAACAGATAAGTCCGTACGGCTAACTTACCTTTCTTTGT	EBS1/δ
BAS4553-343|344 s	TGAACGCAAGTTTCTAATTTCGATTGCATCTCGATAGAGGAAAGTGTCT	EBS2
BAS4597-1794|1795 s	AAAAAAGCTTATAATTATCCTTACGAAACGAGAAAGTGCGCCCAGATAGGGTG	IBS1/2
BAS4597-1794|1795 s	CAGATTGTACAAATGTGGTGATAACAGATAAGTCGAGAAAGATAACTTACCTTTCTTTGT	EBS1/δ
BAS4597-1794|1795 s	TGAACGCAAGTTTCTAATTTCGGTTTTTCGTCGATAGAGGAAAGTGTCT	EBS2
EBS-Universal	AACCGAAATTAGAAACTTGCGTTCA	None

The primers shown were used in PCR to generate a HindIII BsrG1 fragment that was exchanged into the Targetron vectors and retarget the intron to the genomic location indicated in the primer name. The exact nucleotide the intron is designed to insert at is designated by a | at the genomic location indicated above the primer sequences.

### *Bacillus anthracis* genomic DNA isolation

*Bacillus anthracis* was grown overnight and 2 mL were harvested for genomic isolation with the Epicentre Biotechnologies DNA & RNA purification kit (cat. no. MC85200). Final yield was 70 μL at ~1.3 mg/mL.

### *Bacillus anthracis* electroporation

Electroporation conditions described in Koehler et al. were followed with a few modifications [35]. A culture of *Bacillus anthracis* Sterne was grown overnight in BHI. The following day, 1 mL of the overnight culture was used to inoculate 100 mL of BHI supplemented with 0.5% glycerol in a 1000 mL baffled Erlenmeyer flask and incubated at 37°C with shaking at 225 rpm until the culture reached an OD_600_ of ~ 0.6. At this point the culture was chilled on an ice bath and filtered through a filter assembly pre chilled to 4°C and washed with an ice cold electroporation buffer containing 10 mM Hepes pH 7.0 and 10% Glycerol (3×50 mL washes). The washed bacteria were resuspended from the filter using 10 mL of ice cold electroporation buffer, then aliquoted and flash frozen. Aliquots of electrocompetent *Bacillus anthracis* (Sterne) were electroporated with 3 μg of *dam*^*-*^*dcm*^*-*^ plasmid DNA in a 4 mm gap cuvette. All electroporation was conducted on a BioRad Gene Pulser. Electroporation conditions were as follows: 2,500 V, 25 μF, 400 Ω. The time constant for the pulse was (on average) 6.5 ms. After electroporation, the cells were diluted with 1 mL of BHI augmented with 10% glycerol, 0.4% glucose and 10 mM MgCl_2_. The cells were incubated for one hour before plating on LB or BHI agar plates with 20 μg/mL kanamycin or 4 μg/mL erythromycin.

### Generation of targetron insertions

A frozen glycerol stock of *Bacillus anthracis* Sterne transformed with the plasmid vector designed to insert into IS605 was used to inoculate LB augmented with 20 μg/mL kanamycin and shaken at 220 RPM at 37°C overnight. The overnight cultures were used to prepare fresh dilutions by inoculating 100 μL of the overnight culture into 25 mL LB augmented with 20 mg/mL kanamycin and shaken (220 RPM) at 37°C. When the cultures reached an OD_600_ of 0.6, 25 μL of 10 mM CdCl_2_ was added in order to induce the cadmium promoter controlling expression of the targetron. Cultures were induced for 90 min. Serial dilutions of the induced cultures were prepared and 100 μL aliquots taken from the 10^-4^ and 10^-5^ dilutions were plated on LB agar without antibiotic selection. Single colonies were tested for intron insertion via colony PCR using primers flanking the site of intron insertion. A frozen glycerol stock of *Bacillus anthracis* Sterne transformed with the plasmid vector designed to insert into BAS4597 or BAS4553 was used to inoculate LB augmented with 4 μg/mL erythromycin and shaken at 220 RPM at 37°C overnight. The overnight cultures were used to prepare fresh dilutions by inoculating 100 μL into 25 mL LB with 4 μg/mL erythromycin and shaken (220 RPM) at 37°C and grown to saturation. 0.2-0.5 mL of the saturated cultures was spread on LB agar plates containing kanamycin at 20 μg/mL and incubated at 37°C for three days. Any kanamycin resistant colony arising reflects a potential intron insertion and this was verified by colony PCR using primers flanking the site of intron insertion.

### Colony PCR screening

Colonies were assessed for potential intron insertions by conventional PCR. Specific primers flanking the site of chromosomal insertion were designed and synthesized by Integrated DNA Technologies. DNA was amplified using 1 μL of LongAmp *Taq* DNA Polymerase (New England Biolabs), 2.5 μL of 5× LongAmp *Taq* Reaction Buffer, 0.4 μL of 10 mM dNTP Mix (New England Biolabs), nuclease-free water (Ambion), 200 nM of each primer and whole cell template in a final volume of 12.5 μL per reaction. The following cycling conditions were used: initial denaturation at 94°C for 30 sec, followed by 30 cycles of denaturation at 94°C for 30 sec, annealing at 60°C for 45 sec and extension at 65°C for 3 min, with a final extension at 65°C for 10 min. Products were visualized using 1% ethidium bromide (Fisher Bioreagents) on a 1% agarose gel.

## Competing interests

Group II intron gene targeting (“targetron”) technology is subject to U.S. and international patents licensed by The Ohio State University and the University of Texas to InGex, LLC. A.M.L., J.P., the Ohio State University, and the University of Texas are minority equity holders in InGex, LLC. A.M.L. and J.P. may receive royalty payments for commercial use of targetron technology. R.S. has received royalties from The Ohio State University in the past. J.P. is founder of Targetronics, a company sublicensed by InGex, LLC to sell research tools based on targetron technology.

## Authors’ contributions

TJL, RK & DT conceived of the project. RJS designed the experiments along with TJL, JP, AML and AE. RJS, JP, AP, JTW, and PS performed the laboratory work and analyzed the results. RS wrote the manuscript. All authors read and approved the final manuscript.
